# Supplementary material for: Macrophage Depletion Alleviates Immunosenescence in Diabetic Kidney by Modulating GDF-15 and Klotho
Source: Int J Mol Sci. 2025 Apr 23;26(9):3990. doi: 10.3390/ijms26093990 (PMC12071727; doi:10.3390/ijms26093990)
Supplement: Supplementary file 1 [file ijms-26-03990-s001.zip › Figure S1 (Original Uprocessed Immunoblots-Repeats).pdf]

Figure S1. Original unprocessed immunoblots for GDF-15 & Klotho proteins of interest.

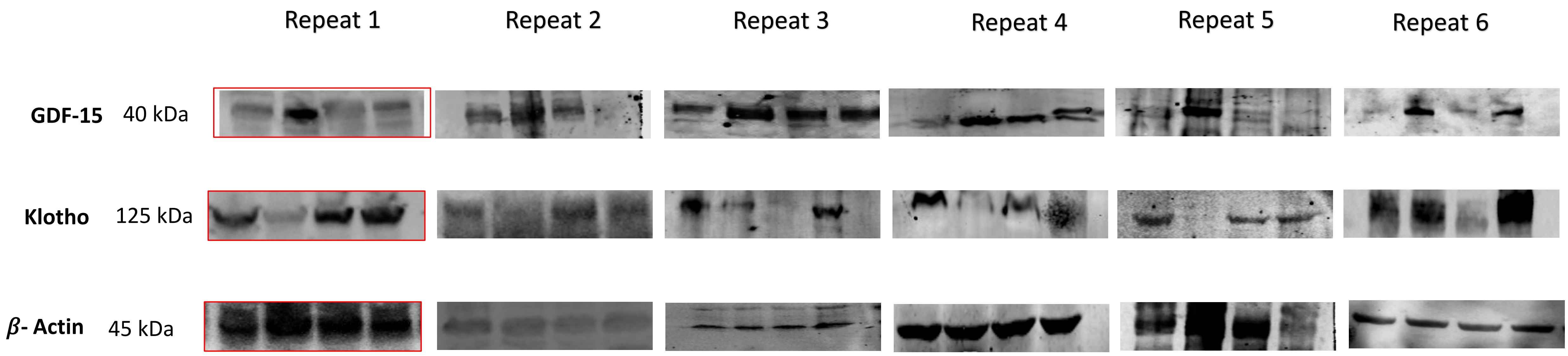

GDF-15

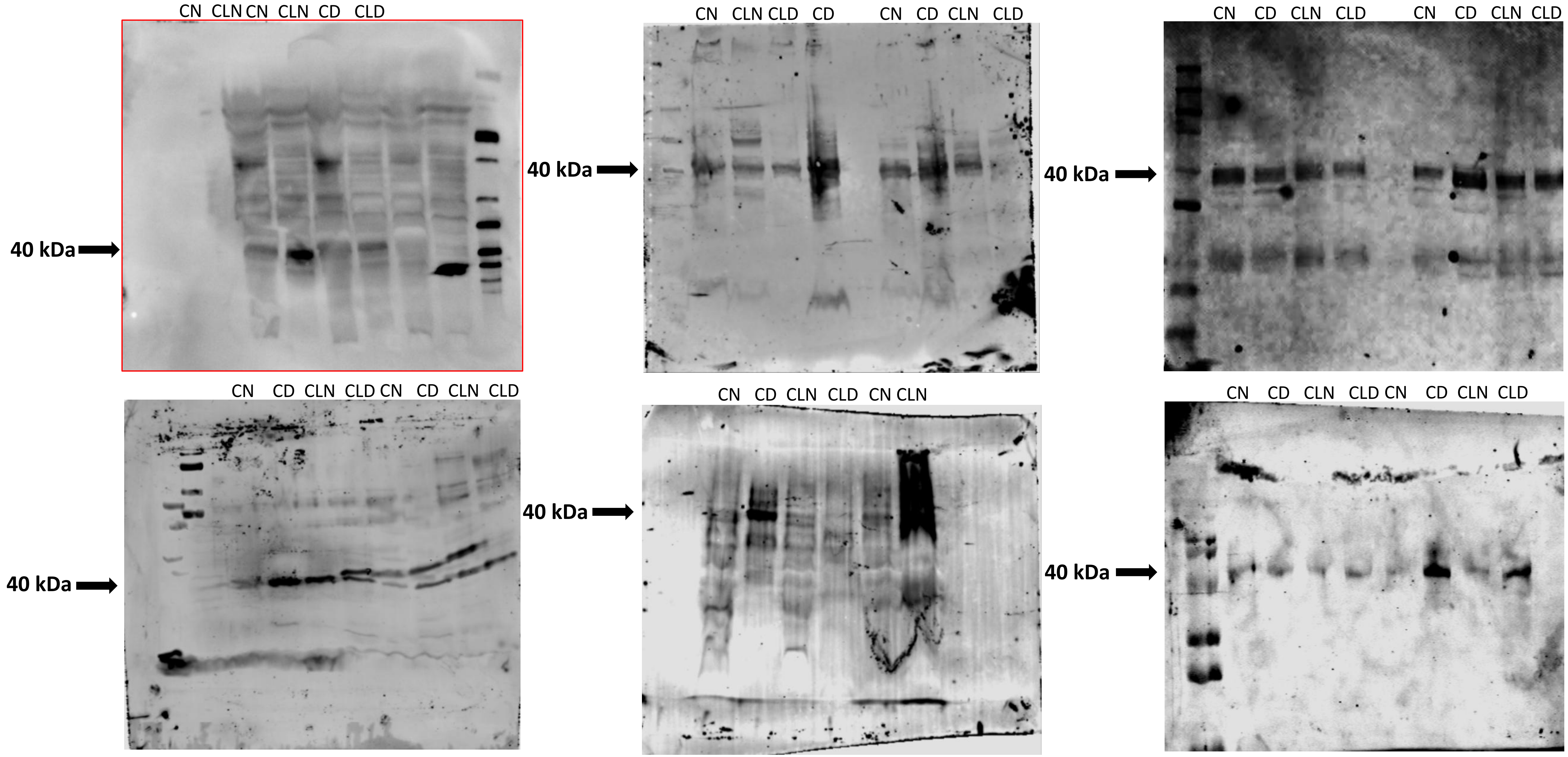

CN CLN CN CLN CD CLD
40 kDa
CN CLN CLD CD CN CD CLN CLD
40 kDa
CN CD CLN CLD CN CD CLN CLD
40 kDa
CN CD CLN CLD CN CD CLN CLD
40 kDa
CN CD CLN CLD CN CLN
40 kDa
CN CD CLN CLD CN CD CLN CLD
40 kDa

Klotho

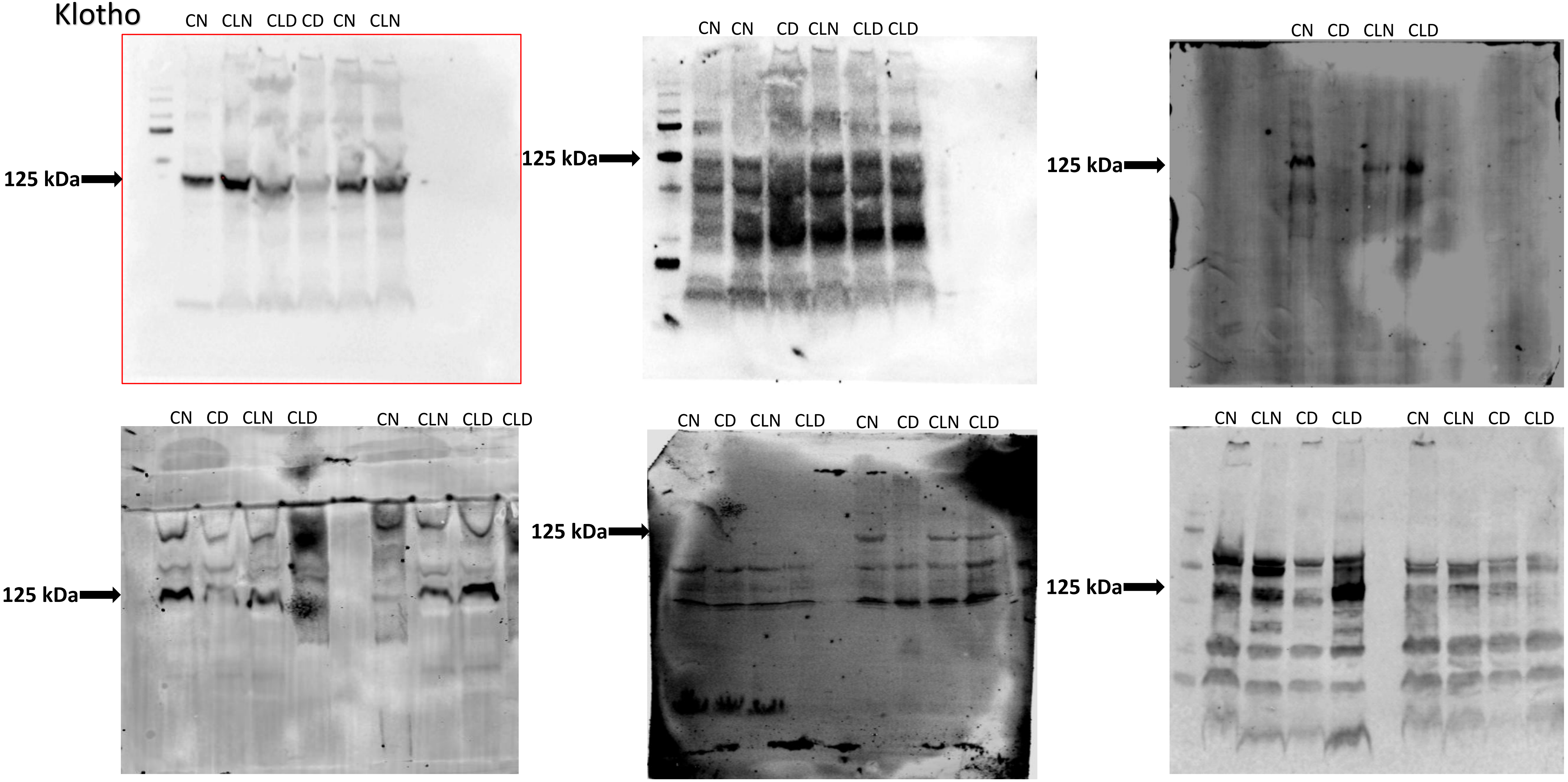
CN CLN CLD CD CN CLN
125 kDa
CN CN CD CLN CLD CLD
125 kDa
CN CD CLN CLD
125 kDa
CN CD CLN CLD CN CLN CLD CLD
125 kDa
CN CD CLN CLD CN CD CLN CLD
125 kDa
CN CLN CD CLD CN CLN CD CLD
125 kDa

β-Actin

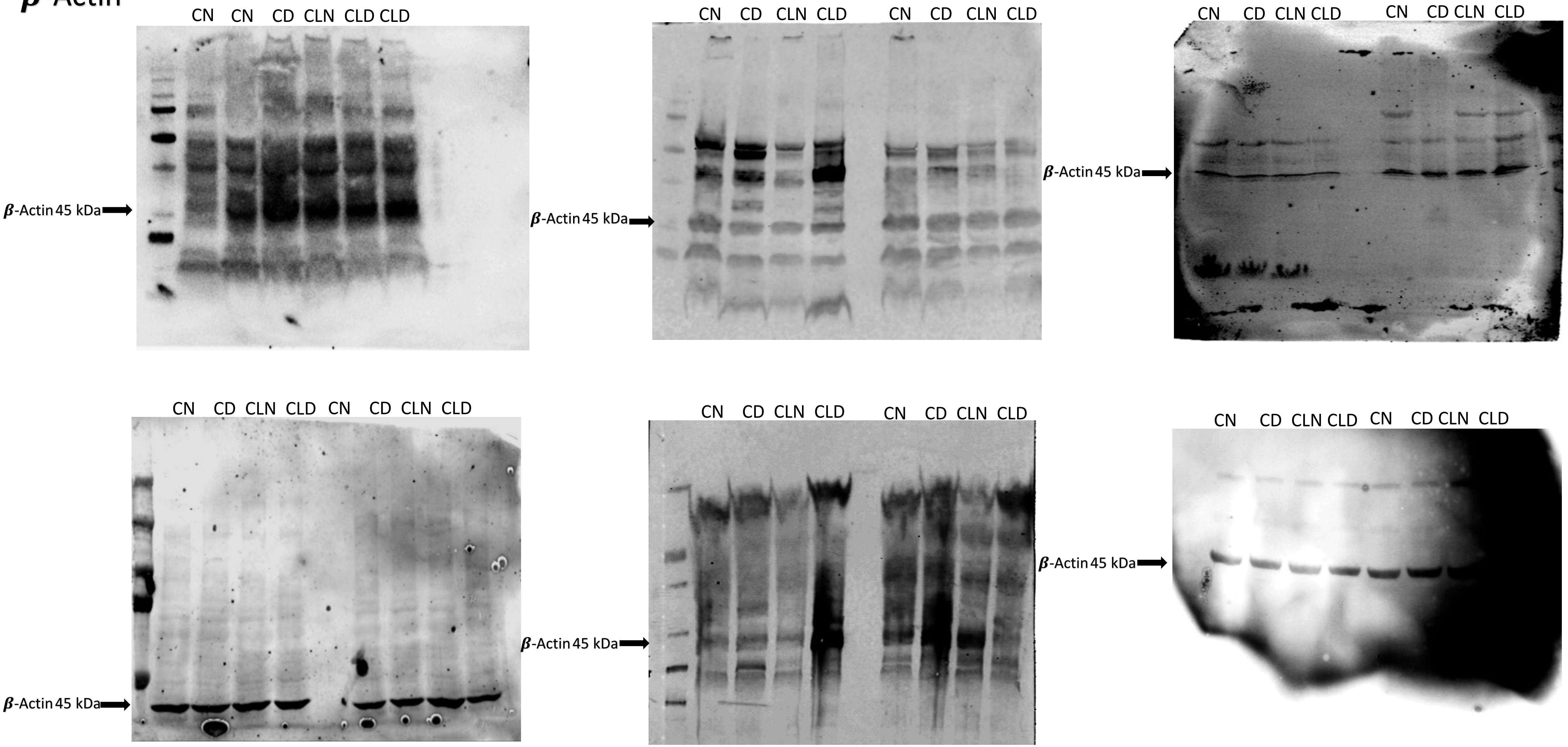
CN CN CD CLN CLD CLD
β-Actin 45 kDa
CN CD CLN CLD CN CD CLN CLD
β-Actin 45 kDa
CN CD CLN CLD CN CD CLN CLD
β-Actin 45 kDa
CN CD CLN CLD CN CD CLN CLD
β-Actin 45 kDa
CN CD CLN CLD CN CD CLN CLD
β-Actin 45 kDa
CN CD CLN CLD CN CD CLN CLD
β-Actin 45 kDa
