# Supplementary material for: Macrophage Depletion Alleviates Immunosenescence in Diabetic Kidney by Modulating GDF-15 and Klotho
Source: Int J Mol Sci. 2025 Apr 23;26(9):3990. doi: 10.3390/ijms26093990 (PMC12071727; doi:10.3390/ijms26093990)
Supplement: Supplementary file 1 [file ijms-26-03990-s001.zip › Table S1 (Original data of biochemical and molecular measurement).pdf]

Table S1 (A). Original data for the effects of Macrophages depletion on metabolic and kidney biomarkers in nephropathy STZ/HFD rats model (glucose levels (mg/dl)).

	CN	CD	CLN	CLD
**Repeat no. 1**	58.44875	338.7097	73.4072	72.09302
**Repeat no. 2**	85.59557	420.2156	82.82548	69.76744
**Repeat no. 3**	81.99446	360.7038	48.19945	156.9767
**Repeat no. 4**	81.89006	416.7116	71.19114	94.76744
**Repeat no. 5**	72.29917	311.3208	62.32687	73.25581
**Repeat no. 6**	68.69806	331.5364	72.02216	70.34884

Table S1 (B). Original data for the effects of Macrophages depletion on metabolic and kidney biomarkers in nephropathy STZ/HFD rats model (Final Body weight (g)).

	CN	CD	CLN	CLD
**Repeat no. 1**	290	332	243	303
**Repeat no. 2**	260	343	258	343
**Repeat no. 3**	276	319	240	330
**Repeat no. 4**	244	286	285	300
**Repeat no. 5**	263	328	189	298
**Repeat no. 6**	289	355	283	265

Table S1 (C). Original data for the effects of Macrophages depletion on metabolic and kidney biomarkers in nephropathy STZ/HFD rats model (kidney/body weight Ratio (mg/g)).

	CN	CD	CLN	CLD
**Repeat no. 1**	2.09424	6.15836	2.23926	2.79310
**Repeat no. 2**	1.28440	8.28863	4.23453	2.17857
**Repeat no. 3**	3.98792	8.13714	2.84047	2.68930
**Repeat no. 4**	2.79412	7.14286	4.23780	2.43636
**Repeat no. 5**	3.04498	8.76543	3.73391	4.12256
**Repeat no. 6**	3.02491	8.75969	2.48201	3.40517

Table S1 (D). Original data for the effects of Macrophages depletion on metabolic and kidney biomarkers in nephropathy STZ/HFD rats model (Albumin (mg/dl)).

	CN	CD	CLN	CLD
**Repeat no. 1**	3.616438356	6.97260274	6.24657534	1.98630137
**Repeat no. 2**	4.059945504	6.95205479	4.99315068	5.43150685
**Repeat no. 3**	4.536585366	8.07534247	6.15068493	4.15068493
**Repeat no. 4**	3.534675615	6.29452055	5.26712329	1.95205479
**Repeat no. 5**	4.831989247	5.39041096	6.47260274	5.34246575
**Repeat no. 6**	4.924657530	5.63013699	4.79452055	2.85616438

Table S1 (E). Original data for the effects of Macrophages depletion on metabolic and kidney biomarkers in nephropathy STZ/HFD rats model (creatinine (IU/L)).

	CN	CD	CLN	CLD
**Repeat no. 1**	1.80487805	6.27586207	3.29411765	2.11764706
**Repeat no. 2**	2.48780488	10.70588240	4.70588235	1.88235294
**Repeat no. 3**	1.60975610	9.00000000	2.82352941	1.88235294
**Repeat no. 4**	4.29268293	3.10344828	3.53333333	1.76470588
**Repeat no. 5**	3.75609756	6.06666667	4.70588235	2.94117647
**Repeat no. 6**	2.46666667	5.29411765	2.55172414	1.66666667

Table S1 (F). Original data for the effects of Macrophages depletion on metabolic and kidney biomarkers in nephropathy STZ/HFD rats model (urea (mg/dl)).

	CN	CD	CLN	CLD
**Repeat no. 1**	6.60660661	11.0476190	4.70588235	4.46666667
**Repeat no. 2**	3.48348348	26.0952381	2.82352941	3.06666117
**Repeat no. 3**	6.54804270	23.4285714	4.82352941	9.11166660
**Repeat no. 4**	5.52552553	21.3333333	4.70588235	4.20000000
**Repeat no. 5**	5.52380952	18.6666667	11.1764706	11.2000000
**Repeat no. 6**	4.12811388	13.4534535	9.28571429	8.76868327

Table S1 (G). Original data for the effects of Macrophages depletion on metabolic and kidney biomarkers in nephropathy STZ/HFD rats model (BUN (mg/dl)).

	CN	CD	CLN	CLD
**Repeat no. 1**	3.08528529	7.40190473	2.19764706	2.19764706
**Repeat no. 2**	1.62678679	12.1864762	1.31858823	1.31858823
**Repeat no. 3**	3.05793594	10.9411428	2.25258823	2.25258823
**Repeat no. 4**	2.58042042	9.96266665	2.19764706	2.19764706
**Repeat no. 5**	2.57961905	8.71733335	5.21941177	5.21941177
**Repeat no. 6**	2.82488300	10.00314000	2.89073000	4.09092000

Table S1 (H). Original data for the effects of Macrophages depletion on oxidative stress biomarkers in nephropathy STZ/HFD rats model (Malondialdehyde (MDA) Kidney tissue levels (U/g)).

	CN	CD	CLN	CLD
**Repeat no. 1**	56.50	126.00	65.25	43.00
**Repeat no. 2**	48.25	146.50	70.00	48.50
**Repeat no. 3**	41.00	156.87	48.75	50.25
**Repeat no. 4**	48.75	155.12	64.50	50.75
**Repeat no. 5**	35.75	166.75	47.00	52.25
**Repeat no. 6**	54.00	143.87	56.00	45.00

Table S1 (I). Original data for the effects of Macrophages depletion on oxidative stress biomarkers in nephropathy STZ/HFD rats model (Superoxide dismutases (SOD) Kidney tissue levels (U/mg)).

	CN	CD	CLN	CLD
**Repeat no. 1**	93.80	14.90	82.60	55.45
**Repeat no. 2**	98.65	32.45	85.80	74.00
**Repeat no. 3**	85.79	18.17	46.20	56.70
**Repeat no. 4**	88.57	32.00	86.30	58.55
**Repeat no. 5**	89.35	23.90	44.40	66.85
**Repeat no. 6**	91.65	31.77	70.70	62.80

Table S1 (J). Original data for the effects of Macrophages depletion on oxidative stress biomarkers in nephropathy STZ/HFD rats model (Glutathione peroxidase (GPX) protein expession (U/mg)).

	CN	CD	CLN	CLD
**Repeat no. 1**	32.08571429	5.771428571	26.54285714	28.85000
**Repeat no. 2**	8.428571429	5.671428571	26.61411571	22.37500
**Repeat no. 3**	30.44285714	10.02857143	20.62857143	21.65000
**Repeat no. 4**	23.94285714	8.104257024	32.75714286	20.00250
**Repeat no. 5**	34.05714286	6.600203700	9.328571429	18.60000
**Repeat no. 6**	22.64285714	5.61428571	26.08501119	22.2750

Table S1 (K). Original data for the effects of Macrophages depletion on inflammatory biomarkers levels in STZ-induced diabetic rats (Interleukin 10 (IL-10) protein expression (pg/ml)).

	CN	CD	CLN	CLD
**Repeat no. 1**	20.533657067	7.6447730810	13.832267648	21.207935361
**Repeat no. 2**	19.187368433	8.9652605144	14.045433901	17.844095272
**Repeat no. 3**	19.411540190	6.9856284427	15.389226107	20.309065789
**Repeat no. 4**	21.207935361	7.8646506361	14.054441977	19.8601351820
**Repeat no. 5**	18.515355552	7.6457730810	12.722634771	17.620508871
**Repeat no. 6**	18.963280439	6.9856284427	14.276698999	18.291518565

Table S1 (L). Original data for the effects of Macrophages depletion on inflammatory biomarkers levels in STZ-induced diabetic rats (Interleukin 6 (IL-6) protein expression (pg/ml)).

	CN	CD	CLN	CLD
**Repeat no. 1**	4.26700	9.79400	4.83100	3.14440
**Repeat no. 2**	4.54900	8.25300	4.64300	3.89200
**Repeat no. 3**	4.73700	8.34900	5.58600	3.79800
**Repeat no. 4**	5.20800	8.54100	7.39100	4.73700
**Repeat no. 5**	6.05900	8.63700	7.48700	5.39700
**Repeat no. 6**	4.19840	9.88408	4.72922	3.01432

Table S1 (M). Original data for the effects of Macrophages depletion on inflammatory biomarkers levels in STZ-induced diabetic rats (Tumour Necrosis Factor alpha (TNF-α) protein expression (pg/ml)).

	CN	CD	CLN	CLD
**Repeat no. 1**	10.590582740	21.787981106	17.084084578	18.020444011
**Repeat no. 2**	16.461064542	23.685119060	15.839017510	16.149919543
**Repeat no. 3**	13.360513962	23.051743173	16.149919543	17.084084578
**Repeat no. 4**	10.038915487	30.396379607	18.645906566	16.77245279
**Repeat no. 5**	16.283460000	32.010849447	17.708079901	16.461064542
**Repeat no. 6**	18.333052804	28.467468435	17.809839960	18.020444011

Table S1 (N). Original data for the effects of Macrophages depletion on Histopathological examination in STZ-induced diabetic rats (Hematoxylin and Eosin stain (H & E) (Macrophages (+) cells (% of 6 fields)).

	CN	CD	CLN	CLD
Repeat no. 1	92	196	62	87
Repeat no. 2	78	249	91	81
Repeat no. 3	73	142	92	36
Repeat no. 4	53	160	55	58
Repeat no. 5	60	373	80	29
Repeat no. 6	61	182	81	39

Table S1 (O). Original data for the effects of Macrophages depletion on Histopathological examination in STZ-induced diabetic rats (Periodic acid-Schiff (PAS) (PAS/macrophages (+) cells (% of 6 fields)).

	CN	CD	CLN	CLD
Repeat no. 1	42	150	70	15
Repeat no. 2	20	90	90	31
Repeat no. 3	33	160	50	16
Repeat no. 4	94	130	60	41
Repeat no. 5	58	200	80	50
Repeat no. 6	29	98	52	23

Table S1 (P). Original data for the effects of Macrophages depletion on Immunohistochemistry examination in STZ-induced diabetic rats (MCP-1 (+) cells (% of 6 fields)).

	CN	CD	CLN	CLD
Repeat no. 1	44	274	127	23
Repeat no. 2	51	124	102	19
Repeat no. 3	21	181	13	95
Repeat no. 4	61	212	33	34
Repeat no. 5	51	143	23	104
Repeat no. 6	42	142	82	43

Table S1 (Q). Original data for the effects of Macrophages depletion on Immunohistochemistry examination in STZ-induced diabetic rats (P16 ^{INK4a} (+) cells (% of 6 fileds)).

	CN	CD	CLN	CLD
**Repeat no. 1**	54.842	201.526	59.245	52.170
**Repeat no. 2**	24.716	187.595	62.830	37.026
**Repeat no. 3**	45.380	191.984	61.949	42.879
**Repeat no. 4**	83.532	159.160	76.052	41.687
**Repeat no. 5**	37.586	147.137	35.585	34.868
**Repeat no. 6**	50.006	151.015	39.150	42.033

Table S1 (R). Original data for the effects of Macrophages depletion on Immunohistochemistry examination in STZ-induced diabetic rats (CD86 (+) cells (%/ of 6 fileds)).

	CN	CD	CLN	CLD
**Repeat no. 1**	35	149	49	31
**Repeat no. 2**	26	123	28	32
**Repeat no. 3**	44	290	64	24
**Repeat no. 4**	53	90	68	47
**Repeat no. 5**	62	123	43	33
**Repeat no. 6**	32	222	51	40

Table S1 (S). Original data for the effects of Macrophages depletion on Immunohistochemistry examination in STZ-induced diabetic rats (CD163 (+) cells (%/ of 6 fields)).

	CN	CD	CLN	CLD
**Repeat no. 1**	330	85	117	166
**Repeat no. 2**	275	65	320	263
**Repeat no. 3**	249	62	181	350
**Repeat no. 4**	128	82	260	245
**Repeat no. 5**	236	92	281	350
**Repeat no. 6**	189	81	222	182

Table S1 (T). Original data for the effects of Macrophages depletion on Protein expression in STZ-induced diabetic rats; the Growth/differentiation factor-15 (GDF-15) protein expression (fold of induction) .

	CN	CD	CLN	CLD
Repeat no. 1	1.000009412	4.938070005	1.177591160	1.012413135
Repeat no. 2	1.000000000	6.011290678	1.024519529	1.108444286
Repeat no. 3	1.000000003	6.408108124	2.069100532	2.769979604
Repeat no. 4	1.009693576	8.077404328	1.568117513	1.329545302
Repeat no. 5	1.021428571	7.682576451	1.052012225	1.043127066
Repeat no. 6	1.000000005	4.545560005	1.022311000	1.004350000

Table S1 (U). Original data for the effects of Macrophages depletion on Protein expression in STZ-induced diabetic rats; Klotho protein (Klotho) protein expression (fold of induction) in STZ-induced diabetic rats.

	CN	CD	CLN	CLD
Repeat no. 1	1.0000001001	0.449657869	1.195770018	1.773793657
Repeat no. 2	1.0000011000	0.433104465	1.088835433	1.078826487
Repeat no. 3	1.0000000010	0.561314408	1.016219034	1.044046646
Repeat no. 4	1.0000000010	0.412234262	1.009620139	1.132807351
Repeat no. 5	1.0001100001	0.292805547	1.155432140	1.107336020
Repeat no. 6	1.0000000400	0.256230000	1.002220000	1.070077000
